# Characterization of miRNAs in Response to Short-Term Waterlogging in Three Inbred Lines of *Zea mays*

**DOI:** 10.1371/journal.pone.0039786

**Published:** 2012-06-29

**Authors:** Zhijie Liu, Sunita Kumari, Lifang Zhang, Yonglian Zheng, Doreen Ware

**Affiliations:** 1 National Key Laboratory of Crop Genetic Improvement, Huazhong Agricultural University, Wuhan, Hubei, People’s Republic of China; 2 Cold Spring Harbor Laboratory, Cold Spring Harbor, New York, United States of America; 3 United States Department of Agriculture – Agriculture Research Service, Robert W. Holley Center for Agriculture and Health, Ithaca, New York, United States of America; USDA-ARS, United States of America

## Abstract

Waterlogging of plants leads to low oxygen levels (hypoxia) in the roots and causes a metabolic switch from aerobic respiration to anaerobic fermentation that results in rapid changes in gene transcription and protein synthesis. Our research seeks to characterize the microRNA-mediated gene regulatory networks associated with short-term waterlogging. MicroRNAs (miRNAs) are small non-coding RNAs that regulate many genes involved in growth, development and various biotic and abiotic stress responses. To characterize the involvement of miRNAs and their targets in response to short-term hypoxia conditions, a quantitative real time PCR (qRT-PCR) assay was used to quantify the expression of the 24 candidate mature miRNA signatures (22 known and 2 novel mature miRNAs, representing 66 miRNA loci) and their 92 predicted targets in three inbred *Zea mays* lines (waterlogging tolerant Hz32, mid-tolerant B73, and sensitive Mo17). Based on our studies, miR159, miR164, miR167, miR393, miR408 and miR528, which are mainly involved in root development and stress responses, were found to be key regulators in the post-transcriptional regulatory mechanisms under short-term waterlogging conditions in three inbred lines. Further, computational approaches were used to predict the stress and development related cis-regulatory elements on the promoters of these miRNAs; and a probable miRNA-mediated gene regulatory network in response to short-term waterlogging stress was constructed. The differential expression patterns of miRNAs and their targets in these three inbred lines suggest that the miRNAs are active participants in the signal transduction at the early stage of hypoxia conditions via a gene regulatory network; and crosstalk occurs between different biochemical pathways.

## Introduction

Land plants, being strictly aerobic, receive freely diffused molecular oxygen from aerial or underground tissues. Oxygen deficiency due to short-term waterlogging (hypoxia) or its complete absence (anoxia) often damages roots under transient or sustained flooding conditions [1]. Long-term waterlogging can cause crop yield losses up to 30% when it occurs early in the season [2], [3]. Maize survival is mainly dependent on its metabolic, physiological and morphological adaptation strategies. In hypoxia, anaerobically induced polypeptides (ANPs) are selectively synthesized, such as aldolase, enolase, alcohol dehydrogenase (ADH) and lactate dehydrogenase (LDH) [4]. Physiologically, anaerobic stress induces alteration of metabolic pathways, leading to the accumulation of metabolites such as ethanol, lactates, and CO_2_, as well as changes in cytosolic pH, reactive oxygen species (ROS) and hormone homeostasis [5], [6], [7], [8].

Small RNAs have been identified as important post-transcriptional regulators of gene expression [9], [10], [11]. Many studies suggest important roles for miRNAs in plant responses to abiotic and biotic stresses such as cold [12], [13], [14], salt [12], [13], [15], [16], heat [13], dehydration [12], [17], oxidative stress [18], mechanical stress [13], pathogen infection [19], and submergence [20]. In addition to studying the effects of stress on miRNA levels, the identification of targets of miRNAs can provide clues about the roles of miRNAs in stress responses [21]. The known targets of post-transcriptional regulation include transcription factors (TFs), suggesting roles of miRNAs as early signaling components that could ultimately lead to broader changes in gene expression following stress.

The response of a plant to hypoxia can be conceptually divided into three stages [22]. The first stage (0–4 h) consists of the rapid induction of signal transduction components, which then activates the second stage (4–24 h), a metabolic adaptation. The third stage (24–48 h) involves the formation of gas-filled air spaces (aerenchyma) in the root [22]. In this paper, we have focused on the first stage because it determines the switch from normal to low-oxygen metabolism and plays an essential role in the survival of seedlings.

To characterize early responses, we used three maize inbred lines having different sensitivities to waterlogging when assayed as seedlings under controlled experimental conditions. We verified previous research work showing that inbred Mo17 is highly sensitive to waterlogging, while Hz32 is relatively tolerant [23], [24], [25], [26]. We also included B73, whose genome serves as the complete reference sequence in maize [27] and found it mid-tolerant. High-throughput sequencing technology [28] was used to identify known and novel miRNAs in the tolerant line at the early stage of waterlogging stress treatment. Putative targets of these miRNAs were predicted by computational methods. We developed expression signatures for a set of miRNAs and their putative targets under waterlogging treatment. Comparisons of these signatures among the three inbred lines provided insight into the role of these factors in waterlogging survival. These observations, along with computational identification of stress and development related cis-regulatory elements in the promoters of the differentially expressed miRNA genes, enabled the construction of a miRNA-mediated regulatory network model. This model will serve as a basis for future research to determine mechanisms of anaerobic stress tolerance in maize.

## Results

### Experimental Design

**Figure 1:**
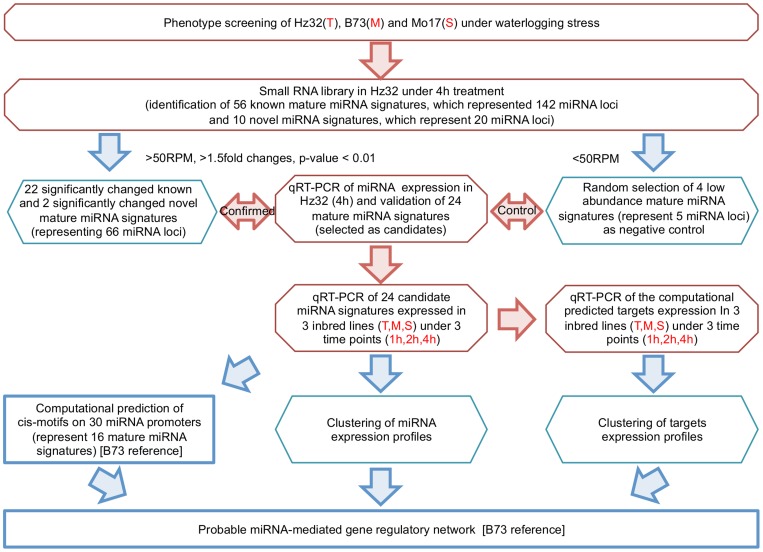
Flowchart of the experimental design. Red octagonal shows all the wet lab experiments. Aqua diamond shows the data analysis method. Blue square shows the computational prediction experiment. T: tolerant line. M: mid-tolerant line. S: sensitive line.

The flow chart of the experimental design is shown in Figure 1. Initially phenotype screening of three inbred lines (Hz32, B73, Mo17) based on the sensitivity to waterlogging tolerance was done. Small RNA library was constructed for the most tolerant line Hz32 under 4 h waterlogging treatment. From the small RNA library, 56 known mature miRNA signatures (representing 142 miRNA loci) and 10 novel mature miRNA signatures (representing 20 miRNA loci) were identified. Out of 56, 22 known mature miRNA signatures (representing 64 miRNA loci) and 2 novel miRNAs (representing 2 miRNA loci) were selected. These 24 mature miRNA signatures, and their computational predicted targets were tested by qRT-PCR assay in three inbred lines that showed different tolerance to waterlogging. Computational approaches were used to predict the stress and development related cis-regulatory elements on the promoters of these differentially expressed miRNAs in the B73 reference maize genome and a miRNA-mediated gene regulatory network was constructed. The results are organized below based on the flow chart of the experimental design.

### Characterization of Tolerance in Three Inbred Lines

**Figure 2:**
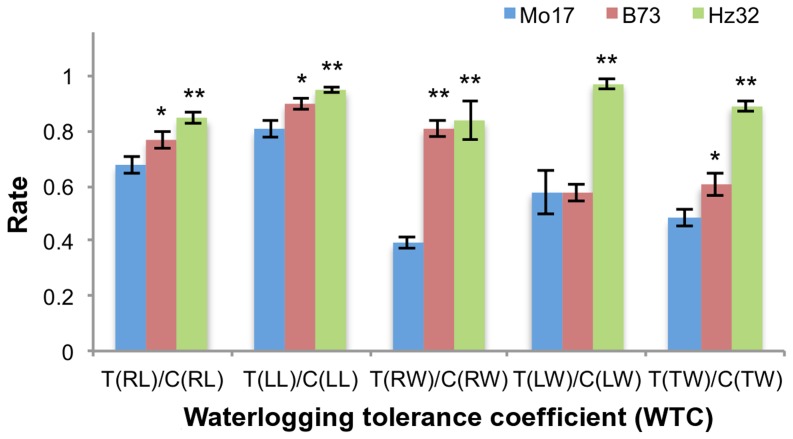
Short-term waterlogging phenotype-screening results of all three inbred lines. T: Waterlogging treatment. C: Control. RL: Root Length. LL: Leaf Length. RW: Root dry Weight. LW: Leaf dry Weight. TW: Total dry Weight. Error bars show the standard deviation. Student t-test was performed on the tolerant line (Hz32) and mid-tolerant line (B73), comparing with sensitive line (Mo17). ** denotes the p-value below 0.01 and * denotes the p-value below 0.05.

Following the screening methods for maize waterlogging tolerance described by Liu *et al* [26] for each of the three inbred lines Hz32, B73 and Mo17, our results showed that the Hz32 is the most tolerant line while Mo17 is the most sensitive and B73 is in the middle range (Figure 2, Table S1). Comparison of the waterlogging tolerance coefficient (WTC) data and the phenotypes with previous studies in Hz32 and Mo17 [23], [24], [25], [26] confirmed the tolerance of these two inbred lines.

### Known miRNA Profiling of Tolerant Inbred Line

As Hz32 is a waterlogging tolerant line, we selected this inbred line for small RNA profiling to get the expression of waterlogging tolerance related miRNAs in Hz32.

From our previous unpublished work on 55 K oligo microarray study of two inbred lines, Hz32 and Mo17 at five different time points of waterlogging treatment (1 h, 2 h, 4 h, 24 h, and 48 h), the gene expression changes reached the peak at 4 h treatment for early responses. Therefore, we picked 4 h as the reference time point for sequencing. To investigate the role of miRNAs under hypoxia stress in Hz32, size fractionated RNAs (18–28 nucleotides) were isolated from Hz32 root tissues subjected to 4 h (signal transduction stage) of waterlogging treatment and control sample (no waterlogging treatment) and sequenced by Illumina high-throughput sequencing [28]. The total number of sequence reads was 9,251,451 for the treatment sample and 9,541,782 for the control sample (Table S2). After removing adapter-only reads and sequences of sizes <18 nt or >26 nt, there were 4,111,022 and 3,802,934 unique sequences (without any duplicates), respectively. The RNA sequences were mainly within a range of 20–24 nt (79.9% and 75.1% for treatment and control samples, respectively), which is the size of most known small RNAs (Figure S1). Overall, the size distribution was similar to previous studies done in angiosperms [29], [30], [31]. Sequence reads that perfectly matched known maize miRNAs [11] were considered. A total of 56 unique mature miRNA signatures, which are specified at 142 miRNA gene loci, matched with known miRNAs from miRBase release 18 [32] (Table S3). Out of 172 miRNA loci described in miRBase release 18 [32], all but 30 were detected in these small RNA libraries.

High-throughput sequencing can both identify miRNAs and measure miRNA expression [33]. The abundance of miRNAs was found to vary greatly within each sample as well as within miRNA families (Table S3). To select putative differentially expressed miRNA genes for detailed study, we set a threshold of 1.5 fold difference in reads per million (RPM) between treated and control and applied a statistical filter (P value <0.01; >50 RPM). Out of 56 unique mature miRNA signatures, 22 known mature miRNA signatures (representing 64 miRNA loci) passed the filtering criteria and were selected for validation using SLRT-PCR (Stem-Loop Real Time PCR) for Hz32 at 4 h waterlogging treatment.

### Detection of Novel Waterlogging Induced miRNAs and their Putative Targets

**Figure 3:**
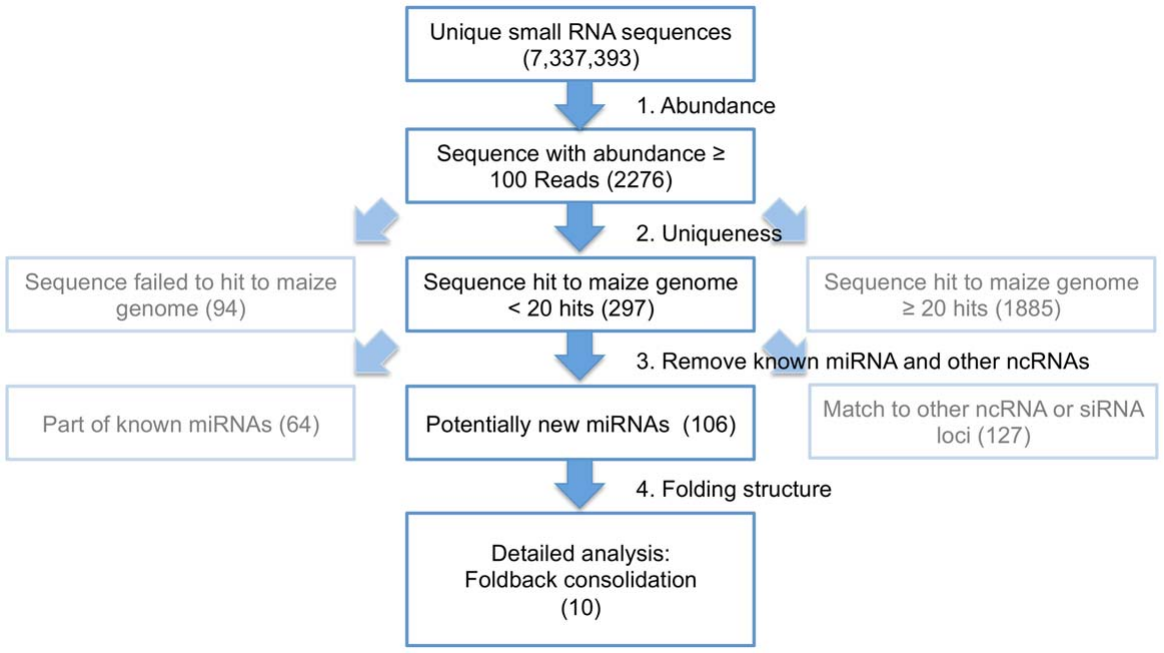
Flowchart of the novel miRNA prediction pipeline. The flowchart shows the filtering criteria to reduce and refine the numbers of small RNAs to the most interesting subset based on the abundance, removal of repeats (uniqueness), removal of known miRNAs, other ncRNAs, and folding structure. 10 unique sequences were identified as potentially novel miRNA candidates. Light blue squares show the filtered out sequences. Blue squares show the sequences that passed the filter.

The total numbers of 7,337,393 unique sequences from the treatment and control libraries (mentioned above) were also used to identify novel miRNAs specific to waterlogging treatment. After applying various filtering criteria such as removing repeats and filtering out the known miRNAs and other noncoding RNAs (ncRNAs), 106 unique sequences were identified as potentially novel miRNA candidates. Out of 106 putative novel miRNA sequences, only 10 miRNA sequences passed the secondary structure evaluation pipeline (Figure 3). These 10 miRNAs, representing 20 miRNA loci, were selected as waterlogging induced novel miRNAs (Table S4) and the putative targets of these miRNA families were predicted using computational target prediction pipeline (Table S5) [11]. To select the differentially expressed novel miRNAs, same filtering parameters were used as mentioned above for known miRNAs. Only 2 novel miRNAs, miRn6 and miRn7, passed the filtering criteria and these miRNAs were selected for validation using SLRT-PCR. Eight putative targets of these 2 novel miRNAs were also selected to preform the qRT-PCR assay (Figure S5).

### Quantitative Validation of Mature miRNA Signatures

**Figure 4:**
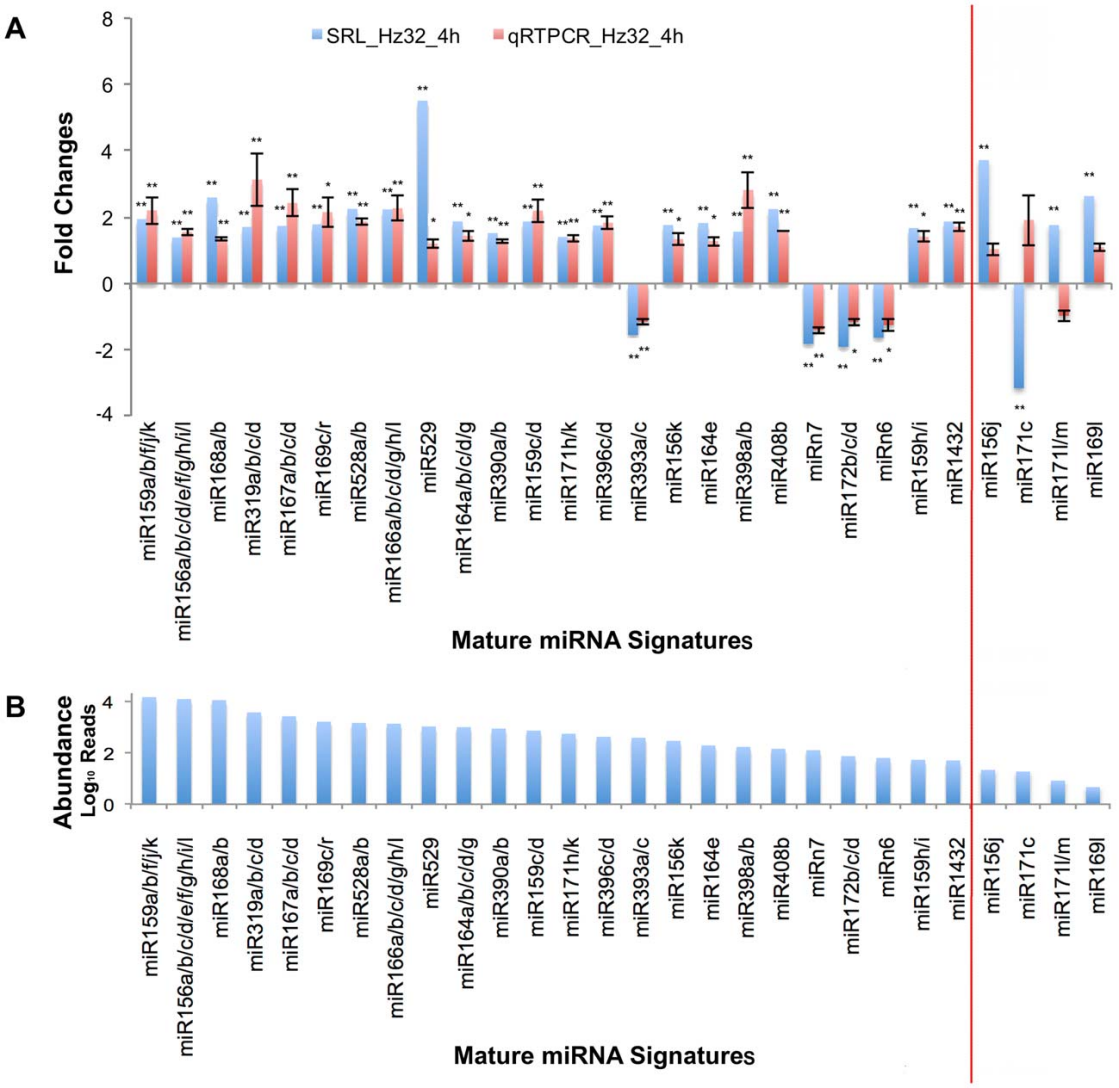
Expression profiles of 24 candidate miRNAs under hypoxia stress. Blue bars show the results of small RNA library in Hz32 under 4 h treatment. Red bars show the results of SLRT-PCR in Hz32 under 4 h treatment at 3 biological replicates. Vertical red line divides the expression profiles of 24 candidate miRNAs with 4 miRNAs (control expression profiles). Error bars show the standard deviation. Chi-square was applied on the small RNA library data and student t-test was performed on SLRT-PCR results. ** denotes the p value <0.01 and * denotes the p value <0.05.

The Quantitative SLRT-PCR protocol [34], [35], [36] was used to measure mature miRNA expression for three biological replicates at 4 h treatment in Hz32. SLRT-PCR was conducted for the 22 mature miRNA sequences and 2 novel mature miRNA sequences. Four low abundance (<50RPM) mature miRNAs (156j, 169l, 171c, 171l/m) were also selected as negative control. Only miRNAs with single amplicon were considered for results. The SLRT-PCR results confirmed most of the miRNAs showed up-regulation under 4 h treatment while only miR172b/c/d, miR393a/c, miRn6, and miRn7 were down regulated in both assays (Figure 4). The low abundance four mature miRNAs were hardly detected by SLRT-PCR, showed either low expression level (<1.2 fold changes; 156j, 169l, 171l/m) or have the opposite expression to the deep sequencing results (171c, 171l/m) (Figure 4). Therefore, these 24 mature miRNA signatures (representing 66 miRNA loci) were selected as candidate genes for further study focusing on temporal and inbred specific expression in the three maize inbred lines under short-term waterlogging conditions.

### Differential Expression of miRNAs in Response to Short-term Waterlogging

**Figure 5:**
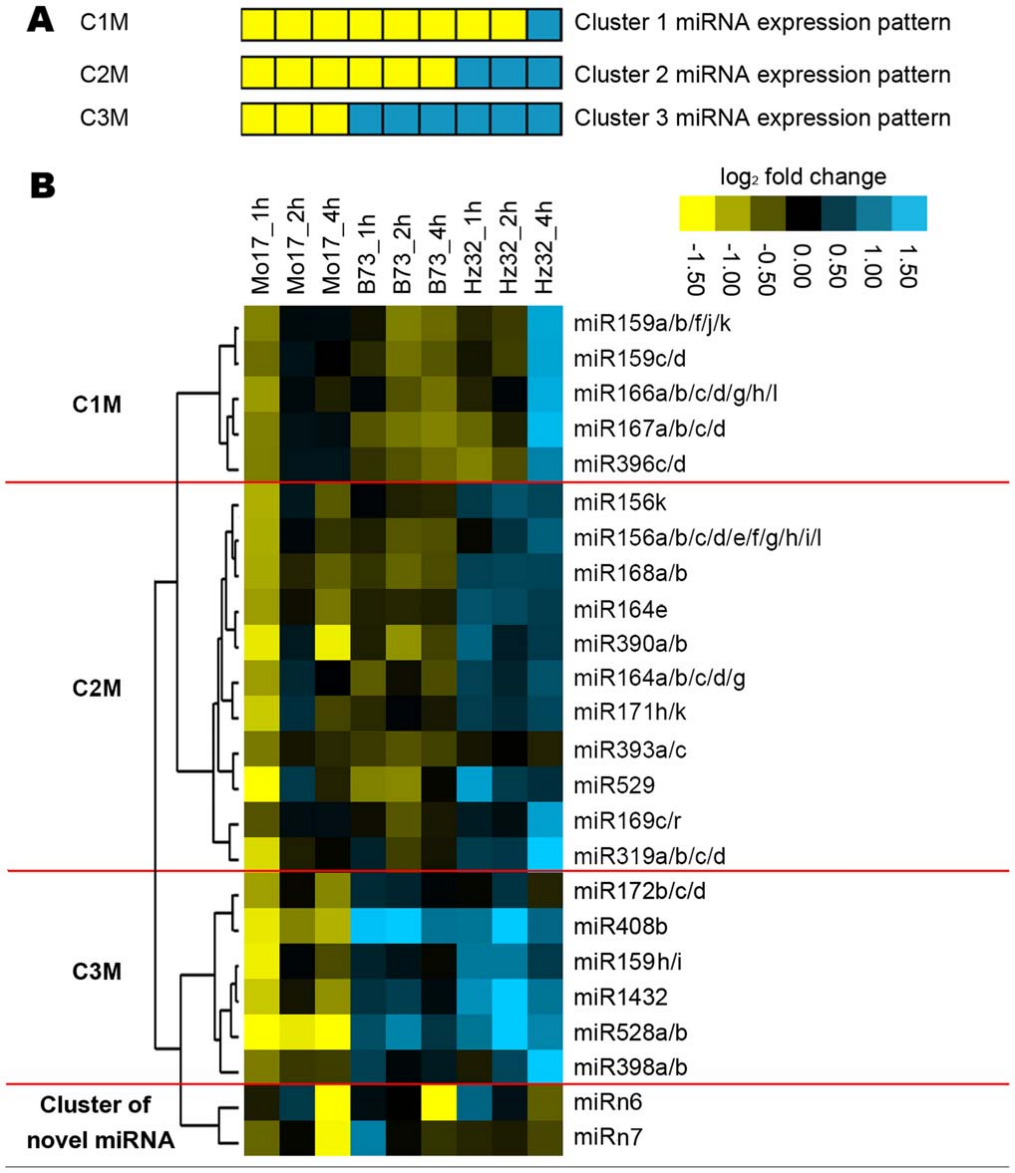
miRNA expression profile by SLRT-PCR and cluster analysis. A: Model of miRNA expression pattern. B: Heatmap of miRNA expression profile. The cluster was done on the basis of log_2_ (expression level in treatment/expression level in control). Yellow shows down-regulation. Blue shows up-regulation.

SLRT-PCR was performed on the selected 24 miRNA signatures under three time point treatments (1 h, 2 h, 4 h) of waterlogging in each of the three inbred lines (Hz32, B73, Mo17) with three biological replicates. Cluster analysis of those miRNAs expression levels among the three inbred lines showed four major clusters (Figure 5).

Cluster 1: miRNAs from families 159(a/b/f/j/k & c/d), 166, 167, and 396 were repressed in all inbred lines except in Hz32 after 4 h treatment. miRNAs from this cluster are involved in flowering or leaf development [12], [37], [38], [39], [40].

Cluster 2: miRNAs from families 156, 164, 168, 169, 171, 319, 390, 393 and 529 were induced in Hz32 and repressed in B73 and Mo17. This cluster is enriched in miRNAs involved in root or shoot development, as well as stress response and tolerance [12], [37], [41], [42], [43], [44], [45], [46], [47].

Cluster 3: miRNAs from families 159(h/i), 172, 398, 408, 528, and 1432 were repressed in Mo17 but were induced in B73 and Hz32. Most of the miRNAs from this cluster are directly involved in stress response [12], [20], [37], [45], [48].

Cluster of novel miRNAs: miRn6 and miRn7 showed a similar pattern among three inbred lines, which are induced at 1 h and 2 h and repressed at 4 h treatment suggesting that these 2 novel miRNAs do not correspond to the differences in the inbred sensitivity to waterlogging.

In the sensitive line (Mo17), the 22 known mature miRNA candidates were generally repressed or showed late, weak, or transient induction. This contrasts with the tolerant line (Hz32) in which these miRNAs generally showed early, strong, and persistent induction. The mid-tolerant (B73) line exhibited a distinct pattern in which some miRNA genes were induced early and persistent while others were not induced or repressed. Cluster 3 miRNAs were induced in both mid-tolerant (B73) and tolerant (Hz32) lines and directly involved in stress response, which indicated that miRNAs from cluster 3 might contribute to B73’s tolerance under waterlogging and be involved in the initial signal transduction. miRNAs from cluster 1 and 2 showed significant differential changes only in the waterlogging-tolerant line (Hz32) and are basically involved in development and stress response. The differential expression of miRNAs among the three clusters showed that these miRNAs were very sensitive to hypoxia and altered at different time points even within a very short time treatment, indicating that miRNAs may be one of the initial signal transduction components in responding to hypoxia at early time.

### Differential Expression of miRNA Targets in Response to Short-term Waterlogging

A large proportion of miRNA targets are proteins that encode TFs required for growth, development, and stress responses [21], [49]. A total of 119 out of 159 (74.8%) computational predicted targets [11] of the known and novel hypoxia-induced miRNAs were assayed by qRT-PCR. Due to challenges associated with sequence paralogs in maize, only 77.3% (92 out of 119) of the predicted targets had single amplicon and these were used for further analysis.

Many of the potential target genes assayed were TFs of the squamosal l-promoter binding protein *(SBP*), myeloblast (*MYB*), Cys3His zinc finger domain (*C3H*), Teosinte branched1/Cycloidea/Proliferating cell factors 1 and 2 (*TCP*), CCAAT-binding factor (*CBF*), auxin response factor (*ARF*), APETALA2-ethylene response factor (*AP2/ERF*), basic region/leucine zipper motif (*bZIP*), growth regulating factor (*GRF*) and no apical meristem (*NAM*) classes.

**Figure 6:**
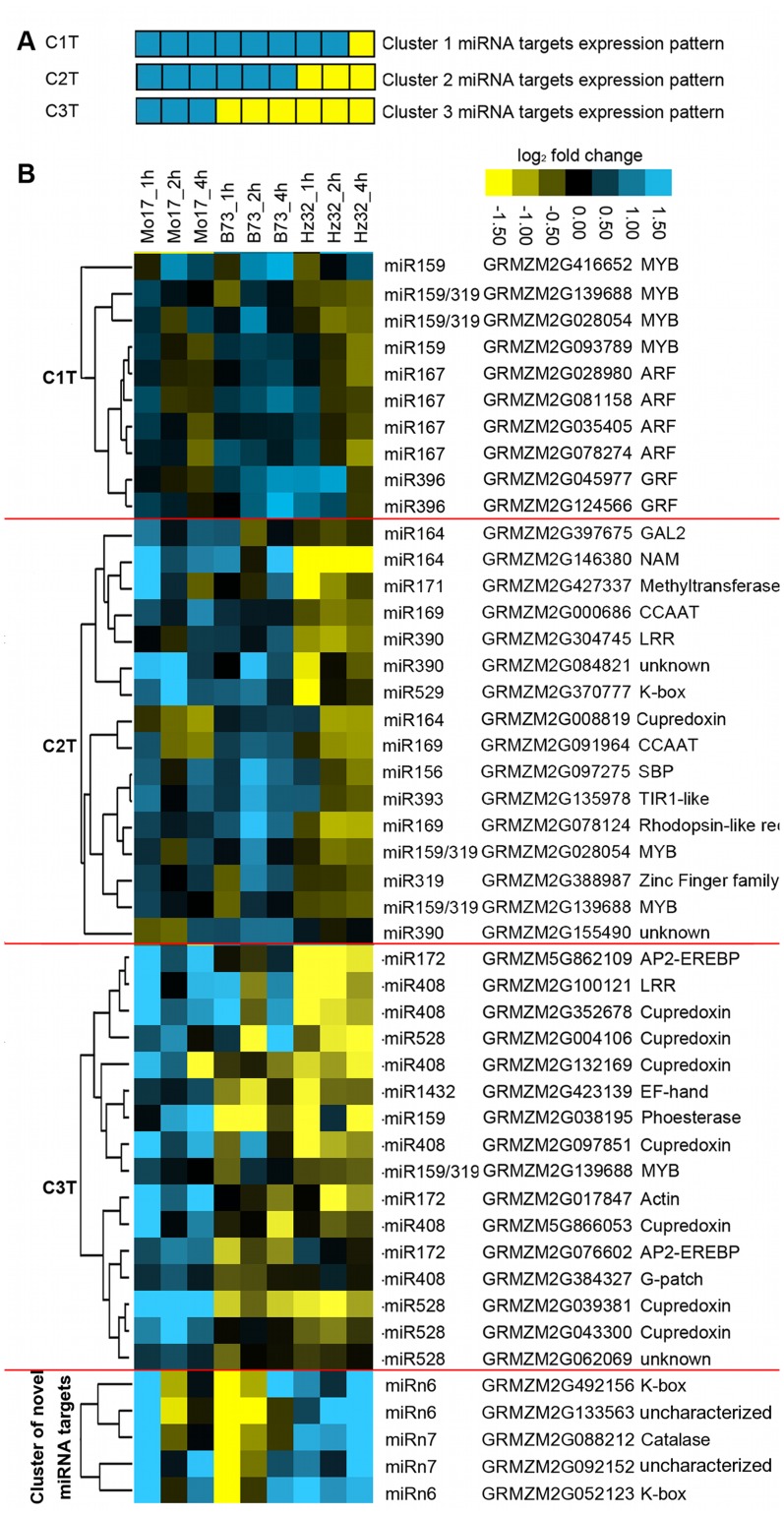
Cluster miRNA targets expression profile. A: Model of miRNA target expression pattern. B: Heatmap of miRNA target expression profile. The cluster was done on the basis of log_2_ (expression level in treatment/expression level in control). Yellow shows down-regulation. Blue shows up-regulation.

The miRNA targets were sub-divided into four groups based on the previously defined miRNA expression clusters (Figures S2, S3, S4 and S5). However, only the targets that showed negative correlation of expression levels with their miRNAs in all three inbred lines considered to be significantly affected by the miRNA and these were selected from each clusters and grouped accordingly (Figure 6).

#### Cluster 1

11 out of 28 (42.9%) miRNA targets (from Figure S2) showed changes in expression that negatively correlated with respect to their corresponding miRNAs in all three inbred lines. These targets belonged to TF families *MYB, ARF* and *GRF*, which are involved in root development, plant secondary metabolism and cell fate [50], [51], [52] (Table S6).

#### Cluster 2

15 out of 41 (36.6%) miRNA targets (from Figure S3) showed changes negatively correlated with their miRNAs in all three inbred lines. These targets belonged to *CBF, K-box, LRR* (leucine-rich repeat), *MYB, SBP, TCP, TIR1-like (F-box)*, *Zinc-Finger* families, methyltransferase and rhodopsin-like receptor, which are involved in signal transduction, protein interaction, meristem growth and plant development [53], [54], [55], [56], [57], [58] (Table S6).

#### Cluster 3

**Figure 7:**
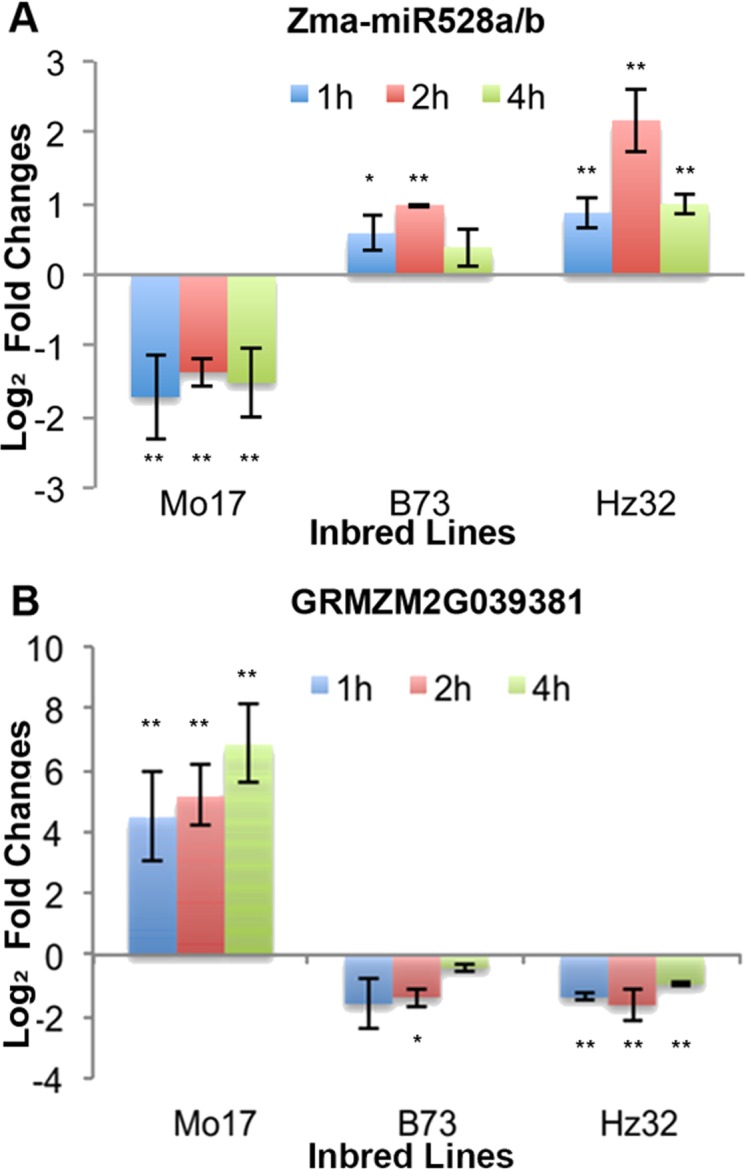
Expression of zma-miR528a/b and one of its targets. A: SLRT-PCR results of miRNA528a/b at 3 biological replicates. B: qRT-PCR results of one of miRNA528a/b targets GRMZM2G039381 at 3 biological replicates. X-axis shows the inbred lines under different time point treatment compared to control. Y-axis shows the expression level. Error bars show the standard deviation. Student t test was performed on qRT-PCR results. ** denotes the p value <0.01 and * denotes the p value <0.05.

16 out of 23 (69.6%) miRNA targets (from Figure S4) showed negative correlation to their miRNAs in all three inbred lines. These targets belonged to *AP2/ERF*, *MYB, cupredoxin*, and *EF hand*. *AP2/ERF* proteins have important functions in the transcriptional regulation of a variety of biological processes related to growth and development, as well as various responses to environmental stimuli [59]. Targets such as *cupredoxin* (miR408 and miR528 targets) are involved in oxidative stress response [60]. An example of miR528a/b and expression of one of its targets, *cupredoxin* (GRMZM2G039381) that shows negative correlation with its miRNA, is shown in Figure 7.

Cluster of novel miRNA targets: Out of eight targets of two novel miRNAs (miRn6 and miRn7), five targets showed negative correlation to their miRNAs in most of the three inbred lines. Based on the interpro [http://www.ebi.ac.uk/interpro/] annotations, two targets have a keratin-like (*K-box*) domain; one has catalase like domain; and remaining two are of uncharacterized function (Figure S5). *K-box* domain is one of domains of *MADS* genes that plays important roles in protein-protein interactions [57]. *MADS* genes are key developmental regulators of vegetative and reproductive development [58]. Catalase plays role in protecting maize roots from toxic effects of hydrogen peroxide (H_2_O_2_) under severe environmental conditions [61].

Based on the expression profiles of miRNA gene target candidates by qRT-PCR, we found fewer than 50% of the targets demonstrated expression levels that were negatively correlated with their miRNAs. There can be two possible reasons. First, recent studies have shown that, in addition to RNA cleavage, miRNAs in plants have widespread translational repressor capability [62], so many of these targets may in fact be regulated through this translational pathway. Second, the miRNA targets may be regulated by more than one miRNA or other TFs, which will lead to different expression pattern. However, the fact that miRNA targets express in different inbred lines in response to waterlogging treatment suggested that miRNAs play a role in regulating transcript expression.

### 5-prime RNA Ligase Mediated Rapid Amplification of cDNA Ends (5′-RLM-RACE) Validation of Selected miRNA Targets

**Figure 8:**
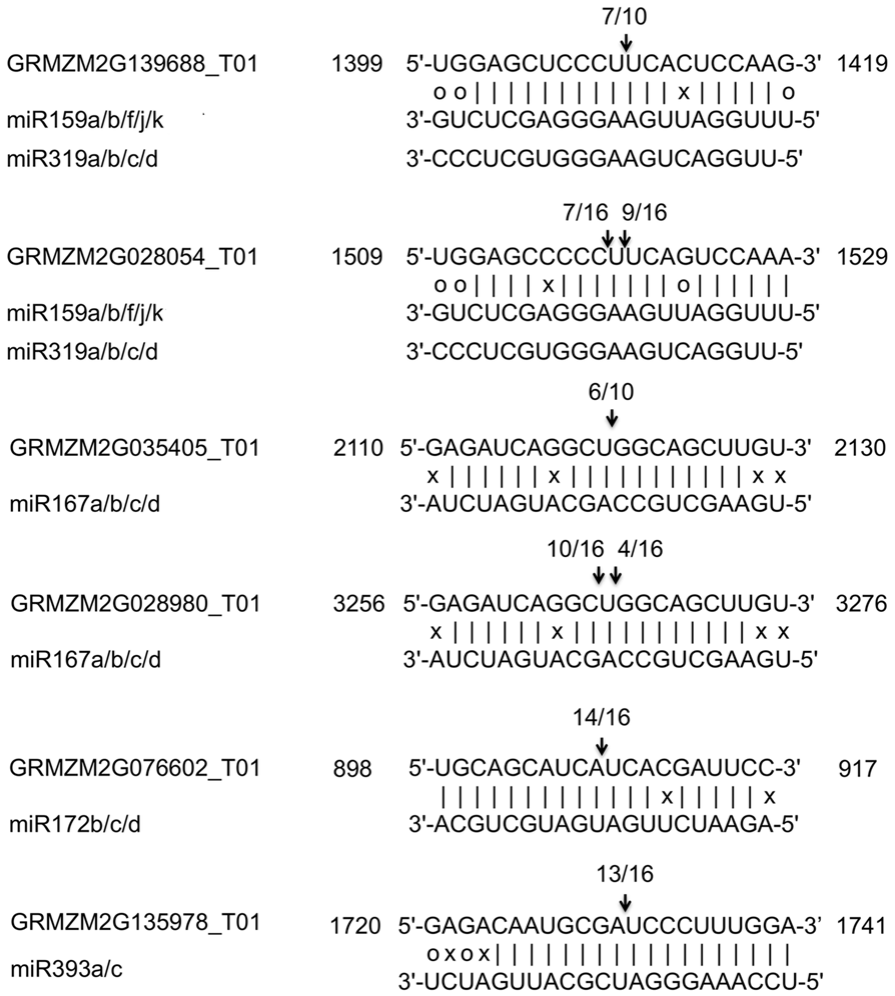
5′-RLM-RACE verification of predicted miRNA targets. Each top strand depicts a miRNA complementary site (targets), and each bottom strand depicts the miRNA. miR159 and miR319 shares the target sequence. Watson-Crick pairing (vertical dashes) and G:U wobble pairing (circles) are indicated. Arrows indicate the 5′termini of mRNA fragments isolated from roots of the maize seedlings, as identified by cloned 5′-RACE products, with the frequency of clones shown. Only cloned sequences that matched the correct target gene and had 5′ ends within a 100 nt window centered on the miRNA complementary site are counted.

5′-RLM-RACE was preformed on thirteen selected miRNA targets to identify their miRNA cleavage site. These 13 targets are targeted by 8 miRNA families (miR159, miR164, miR167, miR172, miR319, miR393, miR528, miRn7) (Table S9), which are active participants in the signal transduction at the early stage of hypoxia conditions. There are six targets (two *GAMYB* targets of miR159/319, two *ARF* targets of miR167, one *AP2/ERF* target of miR172, and one *F-box* (*TIR1-like* gene)), which were validated by the 5′-RLM-RACE (Figure 8). Furthermore, the negative correlation expressions between miRNAs and their targets also imply their relationship, which suggested that those miRNA targets were regulated by their miRNAs.

### Cis-regulatory Element Analysis of miRNA Promoters in B73

To ensure reliable predictions of cis-regulatory elements, also called as transcription factor binding sites (TFBS) or motifs, differentially expressed miRNAs with known transcription start sites (TSS) were used for promoter analysis. To get TSS, we mapped the pre-miRNA coordinates of differentially expressed miRNAs from miRBase release 18 [32] to their *B73 RefGen v1* chromosomal locations [27]. Out of 64 differentially expressed miRNA genes representing 22 known mature miRNA signatures; we could map the TSS only for 30 miRNA genes belonging to 13 miRNA families (Table S10). We further confirmed the coordinates of 16 pri-miRNA genes with published 5′ RACE data [11]. The promoter region [-1000,+500 with respect to TSS] of these 30 differentially expressed miRNA genes were used for computational motif predictions using all experimentally derived position weight matrices (PWMs) from JASPAR core Plantae (http://jasper.genereg.net/) [63] (see methods). We found the occurrence of 18 stress and development associated transcription factor binding sites (TFBS) representing 8 TF families in the selected promoter region of miRNAs (Table S11).

**Figure 9:**
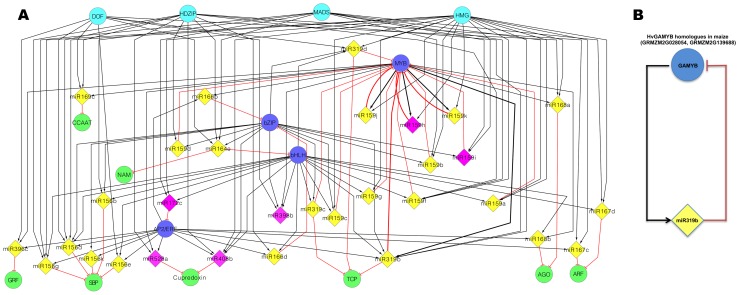
miRNA-mediated gene regulatory sub-network of short-term waterlogging stress in B73. A. TF-miRNA and miRNA-target interactions are shown. B. Probable negative feedback loop between GAMYB and miR319b is shown. Upstream motifs and downstream targets are indicated as circular nodes whereas miRNA are denoted as diamond nodes. Teal color TF nodes represent the upstream motifs, light green nodes represent miRNA targets, whereas royal blue color nodes denote TFs which are involved in both upstream and downstream interactions. Yellow color miRNA shows down regulated miRNAs whereas violet color shows up regulated miRNAs. TF-miRNA interactions are shown as black arrows whereas downstream miRNA-target interactions are shown as red color lines.

Out of 30 differentially expressed miRNAs in B73 that were studied for motif predictions, six miRNA genes (miR159h, miR159i, miR172c, miR398b, miR408b, miR528a) related to cluster 3 are induced whereas rest of the 24 miRNAs genes related to cluster 1 and 2 are repressed under hypoxia conditions. Computational predictions of the motifs in the promoters of the miRNAs of these three clusters showed that these miRNAs were found to have the binding sites of high mobility group proteins (*HMG*) [64], basic helix loop helix (*bHLH*) [65], homeodomain-leucine zipper proteins (*HD-Zip*) [66], [67], *AP2/ERF* [68], *bZIP* [69], [70], [71], *DOF* [72], *MYB* [73], [74], [75] and *MADS* [76], [77], [78] TF families (Figure 9, Table S11 and S12). We have adopted the rigorous filtering methods to predict cis-regulatory elements, but cannot completely eliminate the false positive predictions; therefore these predictions should be experimentally verified for future studies.

#### Cluster 1 and Cluster 2 motifs

The promoter regions of the majority of miRNAs present in cluster 1 and 2 were found to harbor the binding sites of TFs including *HMG-I/Y*, indeterminate protein (*ID1*), *HAT5* (also called as *ATHB1*), ABA insensitive protein (*ABI4*) and plastid envelope DNA binding protein (*PEND*) those belong to *HMG, bHLH, HD-Zip, AP2/ERF*, and *bZIP* TF families respectively (Figure 9, Table S12). Based on the computational analysis of the promoter regions, the cluster 2 miRNAs found to be regulated by more TFs than those from the cluster 1 miRNAs. The binding sites of TFs such as *ATHB5 (HD-Zip), bZIP911 (bZIP), Em-binding protein -1 (EMBP-1, bZIP), HMG-1 (HMG), DOF* and *MYB* TFs families were predominant in cluster 2 miRNA promoter regions whereas *squamosa SQUA (MADS)* binding sites were more present in cluster 1 miRNAs. The putative binding sites of *EMBP-1* found to be enriched in the promoter regions of four miRNA families (miR156b, miR156k, miR164e and miR398b) and these miRNAs were demonstrated to be involved in different parts of plant development and stress [79].

#### Cluster 3 motifs

Expression levels of miR159h/i, miR172c, miR398b, miR408b, and miR528a were decreased in Mo17 but were increased in B73 and Hz32. The putative binding sites of *ABI4* and *HD-Zip* domain binding motifs were present in miR408b and miR528a whereas putative motifs of *bZIP911* and *TGA1A* of *bZIP* family were present in miR398b and miR408b. Additionally, putative binding sites of *HMG*, and *SQUA* TFs were present only in miR172c. The presence of binding sites of *ABI4, HD-Zip* and *bZIP* motifs on the miRNA promoter of this cluster suggests that these TFs are likely helping the induction of miRNA families of this cluster and may be involved in hormonal signal transduction which may repress the lateral root development and inhibit the root or shoot elongation in order to save limited oxygen supply and energy resources.

### miRNA-mediated Gene Regulatory Sub Network

We constructed short-term hypoxia induced probable miRNA-mediated gene regulatory network using Cytoscape [80] (Figure 9). For the upstream miRNA promoter region (TF-miRNA), we predicted the putative binding sites of the TFs based on the experimentally derived PWMs that might be modulating the expression of differentially expressed miRNA genes implicated under waterlogging (Table S11 and S12). For the downstream analysis (miRNA-target), we selected the targets based on the expression profiles of miRNA gene target candidates by qRT-PCR, which were negatively correlated with their miRNAs (Table S8 and S13).

The miRNA targets were functionally annotated based on the TF domains [http://www.ebi.ac.uk/interpro/], therefore individual target genes were labeled based on their TF families. To gain the deeper biological insights from miRNA-mediated pathways, we constructed the regulatory network based on TF families for TF-miRNA and miRNA-TF interactions. miRNA-mediated regulatory interaction network consists of 47 nodes and 133 directed edges (Figure 9). The set of 47 nodes consists of 30 miRNAs (from 13 miRNA families), and 16 TF families. Out of these 16 TF families, TFs of 4 TF families (shown in teal color) putatively bind to the miRNA promoter region whereas target genes belonging to 8 TF families (light green color) are negatively regulated by miRNA genes, and the TFs of remaining 4 TF families (royal blue color) are involved in both upstream (TF-miRNA) and downstream (miRNA-target) interactions (Figure 9A). TF-miRNA interactions are shown as black arrows whereas downstream miRNA-target interactions are shown as red color lines. The miRNA-mediated gene regulated sub-network also shows probable negative feedback circuits between *MYB* and miR319b (Figure 9B). This suggests that TFs of *MYB* TF family might be regulating the expression of these miRNA genes and might also be repressed by these miRNA genes post-transcriptionally.

Based on our computational analysis of homology, we found the *GAMYB* TF (belonging to *MYB*, derived from *Hordeum vulgare HvGAMYB*) binding sites in the promoter region of miR319b. To find the *Zea mays* homologs, the sequence of *Hv*GAMYB was BLASTed and two *GAMYB* genes (GRMZM2G139688, GRMZM2G028054,) were top hits (77% and 68% homology). We found these *GAMYB* target genes among miR319b target genes. Therefore, it can be suggested that *GAMYB* might be regulating miR319b and is also regulated by miR319b post-transcriptionally forming a probable negative feedback loop. We also found the probable feedback loop between three miRNA genes of miR159 (miR159j, miR159h, miR159k), and *MYB* family. However, based on upstream and downstream homology analysis of miR159 (miR159j, miR159h, miR159k), we found *MYB.ph3* binding sites in the promoter region of these three genes of miR159, whereas targets of these genes belong to *GAMYB* family. However, based on this analysis, we can’t assign a negative feedback loop between miR159 and *MYB*, though a probable feedback loop between miR159 and *MYB33* was found in Arabidopsis where feedback involved the down-regulation of *AtMYB33* activity by miR159 and compensatory up-regulation of miR159 by *AtMYB33* [81].

Both miR159 and miR319 are down regulated in B73 under hypoxia conditions. These miRNAs are therefore likely key developmental regulators within the system, and thus, understanding the regulatory control of their upstream regulators will allow us to identify putative cases of negative feedback regulation within the system.

## Discussion

Waterlogging is a complex trait that alters pattern of protein synthesis and metabolic pathways such as ethanol, lactates, and CO_2_, as well as changes in cytosolic pH, ROS, hormone homeostasis, antioxidant enzymes such as superoxide dismutase, ascorbate peroxidase, glutathione reductase and catalase [5], [6], [7], [8]. During the initial hours of hypoxia treatment to maize roots, aerobic protein synthesis is stopped, and gradually anaerobic polypeptides are synthesized leading to the accumulation of alcohol dehydrogenase [4]. Based on our observed differential expression of miRNAs, we hypothesize that these inbred lines might be employing two different miRNA-mediated energy-saving strategies to survive short-term waterlogging stress. The first strategy might be applicable for more sensitive inbred line (Mo17), where the down-regulation of most of the miRNAs would lead to over-expression of downstream targets. As the majority of the targets in our studies are transcriptional activator TFs, probably the rapid involvement of these miRNAs in different signaling pathways are using their entire limited store of O_2_ and sending signals for potentially enhancing the existing developmental regime. However, accelerated O_2_ consumption in this process is a short-term adaptation and might decrease their overall long-term efficiency and fitness and thus making them susceptible to waterlogging stress. An alternative strategy might be applicable for the more tolerant inbred line (Hz32), wherein specific miRNA genes are up regulated thereby reducing their respective target expression levels and causing repression of most of the growth and elongation signaling pathway genes during first few hours. This would shut down unnecessary gene expression and would reserve O_2_ and energy by only inducing genes necessary to withstand the hypoxia stress. The mid-tolerant line, B73 falls somewhere in the middle, with an intermediate response where majority of the miRNA genes are repressed like Mo17 while some miRNA genes are induced as in Hz32.

Expression analysis of targets and promoter analysis in differentially expressed miRNAs subjected to short-term waterlogging stress indicates that the responses might be involved in three major signal transduction pathways, which are ethylene and ABA-dependent signaling pathway, auxin-signaling pathway and *cupredoxin*-mediated oxidative stress responding pathway. To survive under waterlogging, low production of O_2_ might cause ethylene production which promotes death of parenchyma cells that results in the formation of aerenchyma in shoots and roots of a large number of plant species including maize, rice and *Arabidopsis* [82], [83], [84]. ABA is also known as growth inhibitor and the rapid decline in endogenous ABA under waterlogging appears to be due to an increase in ABA breakdown and suppression of its biosynthesis [85]. However, the removal of ABA itself is not the indicator of faster elongation, it merely unlocks ethylene-promoted extension [85]. In *Arabidopsis,* miR159 regulates ABA signaling by targeting *MYB33* and *MYB101* [17]. In our study, *MYB33* homolog (GRMZM2G28054) and *MYB101* homolog (GRMZM2G139688) are targeted by miR159 which have been validated by the 5′-RLM-RACE (Figure 8). The induction of miR159 in waterlogging tolerant line Hz32 suggests miR159 repress the ABA signal by repressing *MYB* proteins.

The second signal transduction pathway is an auxin-signaling pathway that is involved in plant root development. miR164, miR167 and miR393 have been shown to be involved in root cap formation, lateral root development, or adventitious rooting through the auxin signal which is further transduced by their downstream *NAC/NAM*, *ARF* and *F-box* targets respectively [79]. The *NAC (NAM/ATAF/CUC)* family is involved in embryo and shoot meristem development, lateral root formation, auxin signaling, defense and abiotic stress responses [86]. Auxin-induced expression of miR164 and subsequent cleavage of *NAC1* provides a homeostatic mechanism to down-regulate auxin signals for lateral root development in *Arabidopsis* [87]. *NAC-*domain protein (GRMZM2G009892) and *NAM* protein (GRMZM2G146380) were predicted targets of miR164 in our study. qRT-PCR results showed the expression of GRMZM2G146380 was negatively correlated with miR164 in all three inbred lines (Figure 6) while GRMZM2G009892 only showed negative correlation in Hz32 (Figure S4). The induction of miR164 in Hz32 might suggest that miRNA mediated the breakdown of *NAC/NAM* mRNA to attenuate waterlogging signals, which led to reduce embryo/shoot meristem or lateral root production signals at the early stage of waterlogging. The regulatory pathway consisting of miR393 that targets *TIR1*, a negative regulator in auxin signaling [88], may participate in lateral root development in *Arabidopsis* [79]. In our study, miR393 targeted *TIR1-like (F-box)* gene (GRMZM2G135978) (Figure 6 and 8), which was co-expressed with miR393 (Figure 5). Up-regulation of miR393 only in Hz32 may inhibit lateral root production signal at the early stage of hypoxia. miR167 targets *ARF6* and *ARF8* TFs and these *ARFs* were also found to be conserved in rice [89]. The predicted targets of miR167 were *ARF6/8* ortholog genes (Figure 6). *ARF* ortholog genes GRMZM2G028980, GRMZM2G035405 have been confirmed to be cleaved by miR167 (Figure 8) and the expressions of all these ARF genes were negatively correlated with miR167 (Figure 5). Reduction of miR167 at the early stage of waterlogging (except after 4 h treatment in Hz32) suggested the over expression of its target *ARF* and that could induce the adventitious root development signal transduction.

The third major pathway is the *cupredoxin*-mediated hypoxia-responding pathway. *Cupredoxins* are a family of copper proteins with redox activities [90]. *Cupredoxins* are also an important domain in numerous enzymes such as laccases, SOD, multicopper oxidases, metallothioproteins that catalyze several cellular and biological processes ranging from aerobic and anaerobic respiration to iron homeostasis [91]. Both miR408 and miR528 target *cupredoxin*. Both of these miRNAs are highly repressed in Mo17 and are induced in B73 and Hz32. It can be suggested that *cupredoxin* might be important in responding the early signal of waterlogging by mediating electron-transfer or oxidation homeostasis and thus preventing damage to cellular structures.

In addition to these 3 pathways, other miRNAs might be involved in the waterlogging responses such as miR172 that serves as a negative regulator of *AP2,* which is involved in flowering development [92]. In our studies, an *AP2/ERF* domain protein (GRMZM2G076602) that is targeted by miR172b/c/d is reduced in Mo17 but induced in B73 and Hz32 at short-term waterlogging (Figure 6, cluster 3). However, the induced expression level of miR172b/c/d slowly decreases over time from 1 h to 4 h and shows down regulation at 4 h in B73 and Hz32.

To better understand the molecular mechanisms regulating gene expression in response to waterlogging stress, our study has focused on the upstream (TF-miRNA) and downstream (miRNA-targets) interactions and their role in mediating stress responses. The identification of stress and development associated putative cis-regulatory elements in miRNA promoter region will advance our understanding of regulatory networks in which miRNAs play a crucial role. Majority of the miRNAs in three clusters have *HMG, HAT5 (ATHB1), ATHB5, ABI4* and *ID1* binding site motifs. *HMG* family proteins bind to an AT rich enhancer element [64], [93]. These are involved in diverse nuclear processes and biological processes including growth, proliferation, differentiation and death and thus function as central hubs of nuclear function [94]. The functions of *ATHB1, ATHB2* and *ATHB5* belonging to *HD-Zip* family are mainly related to developmental events in response to abiotic stress, ABA and auxin signaling [95], [96]. In *Arabidopsis,* putative motifs of *ATHB1* and *ATHB5* found to be present in the promoter region of hypoxia responsive miRNAs [31]. *ABI4 (AP2/ERF)* is one of the key players that regulate ABA and sugar signaling pathways [68] and probably binds to GCC pathogenesis related promoter element [97]. *ID1*, a basic helix-loop-helix type (*bHLH*) protein, seems to play a key role in regulating the transition to flowering in maize by regulating floral inductive signals [98], [99]. However, other TFs from *bHLH* family such as *AtMYC2* and *ATAIB* function as transcriptional activators and found to be involved in the regulation of ABA signaling in *Arabidopsis* [100].

TFs like *bZIP911, EMBP-1, TGA1A* belonging to basic domain leucine zipper (*bZIP*) family were found enriched in cluster 2 and 3 miRNAs. *bZIP 911* is known for activating ABA-responsive genes via binding to ABA-response element (*ABRE*) [101], [102], [103], [104]. *EMBP-1* is expressed in late embryogenesis and binds to *G-box* (CCACGTGG) motif on the promoter region [68], [101] whereas *TGA1A*, induced by auxin and responsible for root specific expression in tobacco, binds to *C-box* (TGACGTCA) motifs [68], [105]. The computational promoter analysis on up regulated genes in *Arabidopsis* showed the overrepresentation of *G-box* related sequence under waterlogging conditions [106]. The overrepresentation of the *ABRE, G-box* related *and C-box* related motif suggests a potential cross talk among different signaling transduction pathways under waterlogging stress.

TFs from *MYB, bZIP, AP2/ERF* and *bHLH* families that bind to the miRNA promoter region are also negatively regulated by miRNAs (Figure 9). Based on their interactions and network analysis, the system might be employing these TFs as key developmental regulators. *Arabidopsis* response regulator 10 (*ARR10*), a type of *MYB*, was suggested to play an important role in the cytokinin signaling in roots [107] whereas *GAMYB (MYB)* plays role in GA signaling [108], [109]. In *Arabidopsis AtMYB2,* found to be induced by hypoxia [22], [110], dehydration, salt stress and exogenous ABA [111]. This TF binds specifically to the *GT-motif* of anaerobic response element *ADH1* [22], [110]. In wheat, a *MYB* TF called *TaMYB1* was reported to be overexpressed in roots under waterlogging conditions [112] suggested the involvement of *MYB* in low oxygen signaling pathway and helps the survival of wheat plants under anaerobic conditions.

miRNA-mediated regulatory sub-network based on miRNA-TF interactions suggests that one miRNA may be regulated by many TFs. The network also shows probable negative feedback loop between *MYB* and miR319b and *MYB* and miR159. miR319 and miR159 are highly conserved miRNAs and are involved in plant growth, morphogenesis and reproduction [113]. However, these putative interactions need to be confirmed by experimental data. We believe that our computationally predicted miRNA-mediated sub-network will be useful for future research studies and inspire research efforts aiming to better understand miRNA mediated regulatory mechanisms.

Based on our studies, we propose a model of the regulation of differentially expressed miRNAs genes for sensitive, mid-tolerant and tolerant inbred lines under waterlogging stress. miR159, miR164, miR167,miR393, miR408 and miR528, which are involved in root cap formation, lateral root development, root/shoot elongation and plant cell detoxification by scavenging the reactive oxygen species and thus protecting damage to cellular structure were induced under short waterlogging conditions in waterlogging tolerant line Hz32 and repressed in waterlogging sensitive line Mo17. In the tolerant line, the induction of miR159 might regulate ABA signal transduction pathway by repressing their *MYB* targets; induction of miR167 represses *ARF* that has been demonstrated to be involved in lateral root development, or adventitious rooting through the auxin signal [79]. miR164 regulates root or shoot development by repressing *NAC/NAM* proteins whereas miR393 is negative regulator of *TIR1-like (F-box)* and the cleavage of *F-box* gene down-regulate auxin signals. miR408 and miR528 target *cupredoxin*, which are involved in oxidative stress response signal by mediating electron-transfer or oxidation homeostasis and protecting cellular structures. Thus, in the tolerant line, the repression of hypoxia response signals at the early stage of waterlogging may suppress the signals for aerenchyma formation, coleoptiles growth, lateral root development and protection against oxidation to save limited oxygen and energy. Whereas, in waterlogging sensitive line, the repression of these miRNAs induces the overexpression of target genes, which will transduce hypoxia responding signals that will increase the signals for aerenchyma formation, coleoptiles growth, lateral root development and cell detoxification at the very beginning of waterlogging, which will lead to rapid consuming limited oxygen and energy. In B73, the mid-tolerant line, miR159, miR408 and miR528 (similar to Hz32) are induced while miR164, miR167 and miR393 (similar to Mo17) are reduced, suggested the repression of ABA and *cupredoxin* signals and the induction of auxin signals at the initial stages of waterlogging.

In conclusion, our study suggests that the early response to waterlogging is not linear and crosstalk takes place between different biochemical pathways. The signal transduction pathways regulate different plant hormone cascades, provides significant clues for understanding the regulation of gene expression and metabolic adaptation under hypoxia stress. At early hours of waterlogging, plants sense the lack of oxygen around the root system and trigger initial changes to gene expression, and over time at later stage, signal transduction pathways are activated to provide the morphological and metabolic adaptation.

## Materials and Methods

We selected the waterlogging-tolerant maize line, Hz32, the mid-tolerant line, B73, and the waterlogging-sensitive line, Mo17, and used them for this study. Seeds of three inbred lines were sown in sand filled 3-inch pots (8 seeds per pot). The day/night temperatures during seedling development ranged from 25°C to 33°C that was similar to field temperatures during maize seedling stage development [24]. Experiment design is shown in Figure 1.

### Waterlogging Treatment

Waterlogging treatment was given to eight two-week-old plants of each of three maize inbred lines by transferring to a container that filled with water 2–3 cm above the sand surface [23], [24], [25], [26] subjecting these plants to short-term waterlogging (1 h, 2 h and 4 h). The controlled plants were grown in the same condition without waterlogging treatment. Just after treatment, plants were removed from the container. The roots were cleaned and immediately frozen in liquid nitrogen for further biochemical and molecular studies.

### Waterlogging Phenotype Screen

Waterlogging tolerance coefficient (WTC) was used to measure the waterlogging tolerance of three inbred lines that were subjected to stress [26]. The WTC of samples was calculated using the following formula:

WTC = the mean value of treated seedling/the mean value of control seedlings.

Where the mean value of treated seedling is the measurement values of leaf length (LL), root length (RL), leaf dry weight (LW) and root dry weight (RW) of waterlogged plants. The total dry weight (TW = LW+RW) was used to calculate the tolerance of the inbred lines.

### RNA Extraction

Frozen root tissue was ground into a fine white powder. Total RNA extraction was performed using Trizol (Invitrogen, Carlsbad, CA) following manufacturer’s instructions.

### High Throughput Sequencing

High-throughput sequencing of small RNAs (18–28 nt) was done from total RNA by size fractionation and sequenced by Cold Spring Harbor Laboratory Woodbury Genome Research Center (Woodbury, NY, USA) using high-throughput pyrosequencing technology developed by Illumina, using an Illumina 1G Genome Analyzer (Illumina, San Diego, CA) [28], following the manufacturers instructions. All small RNA sequences have been deposited in the Gene Expression Omnibus in GenBank under the identifier (GSE32983).

### Data Analysis of Small RNAs

Ilumina sequencing data were received in FASTQ format with all the reads shown as 36-mers prior to the removal of the 3′ adapter sequences. A custom script was used to trim the adaptor sequence from each sequence. We used the Vmatch large-scale sequence analysis software (www.vmatch.de) to map unique consensus sequences tags to the maize reference genome (*B73 RefGen v1* [27]). A summary of the origin of the small RNA fragments can be found in Table S2. Reads that mapped with known maize miRNA [11] from miRBase release 18 [32] were selected.

### Prediction of Waterlogging Induced Novel miRNAs and their Targets

The unique sequence reads obtained after combining waterlogging treatment and control libraries with appropriate abundance (≥100 reads, around over 10RPM) were selected and mapped with the maize reference genome (*B73 RefGen v1* [27]). Those sequences that overlapped with repetitive elements and transposable elements were removed. Further, the sequences that matched with known maize miRNAs or other ncRNAs with two mismatches were also removed. miRcheck (http://web.wi.mit.edu/bartel/pub/software.html) and RNAfold (http://www.tbi.univie.ac.at/~ivo/RNA/RNAfold.html) were used to predict the miRNA secondary structures [37]. For the target prediction of novel miRNAs, the miRNA target transcript prediction pipeline was developed using Vmatch [www.vmatch.de] and indexed using mkvtree. These mature miRNA sequences were reverse complemented and matched against the indexed maize transcript database with the parameters relaxed to allow up to six mismatches [11]. The matched miRNA target transcripts were filtered by applying empirical rules defined by Schwab *et al.* [114]. Perfect matches were given a score of 0, and all other mismatches were scored 1. Only one mismatch score was allowed between positions 2 to 12 inclusive. However, no mismatches were allowed at position 10 and 11 and no more than 2 consecutive mismatches were allowed after position 12. A maximum of four mismatches was allowed across the length of the mature miRNA.

### Statistical Analysis of miRNA Frequency

To determine whether differences in miRNA frequency between control and waterlogging-treated samples were significant, a χ-square test was performed using total sequence numbers as previously described by Qiu *et al.* [115]. The candidate mature miRNA signatures were selected after applying the filter (P value <0.01; cut off over 1.5 fold; reads >50 reads per million (RPM)) and were selected for validation using SLRT-PCR.

### Validation of Mature miRNA Expression by Stem-loop Real Time PCR (SLRT-PCR)

The stem-loop reverse transcriptase primer for each miRNA consisted of a selfed stem-loop sequence (GTCGTATCCAGTGCAGGGTCCGAGGTATTCGCACTGGATACGAC) with the specificity conferred by a six nucleotide extension at the 3′ end, which is complementary to the last six nucleotides at the 3′ end of mature miRNA [34]. The RT reactions were performed on 100 ng RNA using Superscript III (Invitrogen, Carlsbad, CA) and carried out according to the manufacturer’s instructions. For each RNA sample, two cDNA reactions were performed. The first had all miRNA stem-loop RT primers (Table S6), and the second had 18s ribosome RNA primer (Table S7) for internal control [116]. The reverse transcription product was amplified using a miRNA-specific forward primer and a universal reverse primer on a Rotor-Gene 6000 (QIAGEN, Valencia, CA). Reactions were performed on three independent biological replicates.

### Quantitative Reverse Transcriptase PCR (qRT-PCR) of miRNA Target Gene Expression

The expression of experimentally determined and computational predicted target genes were assayed by qRT-PCR [11]. The reverse transcription product was amplified using gene-specific primers that generated amplicons that overlapped the known or predicted cleavage site (Table S8). Reactions were performed in triplicate on a Rotor-Gene 6000 (QIAGEN, Valencia, CA). Data was normalized to 18S RNA (Table S7) and analyzed using a comparative quantification procedure [115]. Only amplicons confirmed to be a single product by the melt curve from qRT-PCR were used for analysis.

### 5′-RLM-RACE Validation of miRNA Targets

Poly (A) mRNAs from the total RNA of maize roots were purified using mRNA purification kit (TaKaRa, Dalian, China). An RNA adapter was directly ligated to 100 ng of mRNA using a T4 RNA ligase (Invitrogen, Carlsbad, CA). Ligated mRNAs were reverse transcribed using Superscript III reverse transcription kit (Invitrogen, Carlsbad, CA) according to the manufacturer's instructions. 5'-RLM-RACE was performed on the selected maize targets [117]. Initial PCR reactions were done with an adapter primer and complementary gene specific primers. The nested PCR was performed with the nest adapter and the nest gene specific primers. The resulting PCR products were gel purified, cloned by TOPO TA cloning kit (Invitrogen, Carlsbad, CA) and sequenced.

### Cluster Analysis of the Gene Expression

A custom R clustering program was developed for cluster analysis. Two R packages “Cluster” and “mclust” were used for hierarchical cluster. “heatmap.2″ from the R package “gplots” was used for drawing the heat map and the gene tree (Figure 5, 6, S2, S3, S4 and S5).

### Cis-regulatory Element Analysis of miRNAs Promoter

Most miRNA genes, like protein-coding genes, are transcribed by RNA polymerase II (Pol II) that results in 5′ capped and poly (A)-tailed transcripts called primary miRNAs (pri-miRNAs) [118], [119], [120]. Therefore, it is reasonable to assume that the promoters of miRNA genes might be located within the upstream region of pri-miRNAs. However, only the pre-miRNA sequence information is available in the public database such as miRBase [32]. To get TSS, the pre-miRNA coordinates of differentially expressed miRNAs were mapped to their *B73 RefGen v1* chromosomal locations [27]. Only differentially expressed miRNAs with known TSS were used for promoter analysis. Putative cis-regulatory elements were predicted only for the B73 inbred line due to availability of its reference genome. For the other two inbred lines Hz32 and Mo17, promoter sequences were not available due to lack of their respective genome assemblies. Cis-regulatory motifs were computationally predicted in the promoter region of 1000 bp upstream and 500 bp downstream with respect to TSS of the pri-miRNA genes. Search Tool for Occurrences of Regulatory Motifs (STORM) in a Comprehensive Regulatory Element Analysis and Detection (CREAD) suite of tools [121] was used to identify putative transcription factor binding sites using known experimentally derived position weight matrices from JASPAR core Plantae (http://jasper.genereg.net/) [63]. Custom scripts were developed to reduce false positive signal detections. The putative cis-regulatory elements those were over-represented (p-value <0.001) in the promoter sequences of miRNAs as compared to random genomic sequences; and had a frequency of three or more occurrences per miRNA promoter sequences were considered. The p-value was obtained after adjusting for multiple testing by applying Bonferroni correction [122]. Cytoscape [80] was used for constructing and analyzing regulatory network.

## Supporting Information

Figure S1 **Size distribution of sequenced reads of control and 4 h waterlogging treated roots in Hz32.** X-axis shows the size of the reads of the sequencing fragment. Y-axis shows the raw reads of each fragment. Red bar shows the results of control sample. Green bar shows the results of 4 h treatment sample.
(TIF)

Figure S2 **Cluster 1 miRNA targets expression profile.** The cluster was done on the basis of log_2_ (expression level in treatment/expression level in control). Yellow color shows down-regulation. Blue color shows up-regulation.
(TIF)

Figure S3 **Cluster 2 miRNA targets expression profile.** The cluster was done on the basis of log_2_ (expression level in treatment/expression level in control). Yellow color shows down-regulation. Blue color shows up-regulation.
(TIF)

Figure S4 **Cluster 3 miRNA targets expression profile.** The cluster was done on the basis of log_2_ (expression level in treatment/expression level in control). Yellow color shows down-regulation. Blue color shows up-regulation.
(TIF)

Figure S5 **Cluster of novel miRNA targets expression profile.** The cluster was done on the basis of log_2_ (expression level in treatment/expression level in control). Yellow color shows down-regulation. Blue color shows up-regulation.
(TIF)

Table S1 **Grading of maize seedling of 3 maize inbred lines by phenotypic chlorosis under waterlogging stress.** The grades from 1–5 show the tolerance of the maize inbred lines. Each grade has its own description.
(XLSX)

Table S2 **Summary of small RNA sequencing data.** Total reads are the raw reads from the sequencing library. Reads containing N are the reads containing uncertain nucleotide (N = A/T/C/G). Reads without N are the reads without uncertain nucleotide. Adaptor only reads are the reads that only have adaptor sequence. Reads with small insert are the reads length below 18 nt after removing the adapter sequence. Reads with large insert are the reads length over 26 nt after removing the adapter sequence. Filtered reads are the reads that passed all the filters described above. Unique sequences are the unrepeated sequence of the filtered reads.
(XLSX)

Table S3 **Known miRNAs present at 4 h waterlogging and control treatment in Hz32 roots.** First column is the miRNA id. The second column is the read per million (RPM) of the Hz32 control library. The third column is the RPM of Hz32 at 4 h treatment library. The last column is the relative change. Chi-square test is applied to the relative change and * denotes the p value <0.01.
(XLSX)

Table S4 **Novel mature miRNA and their precursor with genome coordinates.** The table gives the information of the predicted novel miRNA such as genome coordinate, mature sequence and pre-miRNA sequence.
(XLSX)

Table S5 **Targets of novel miRNAs with interpro annotation.** List of novel miRNA targets with interpro id and description. MM: miss match.
(XLSX)

Table S6 **miRNA SLRT-PCR primers and their target function.** The first column is the miRNA id. The second column is the gene specific forward primer for the SLRT-PCR. The third column is the stem loop primer for the first strand cDNA amplification. The last column is the predicted miRNA targets annotation. Universal reverse primer is for the SLRT-PCR reaction.
(XLSX)

Table S7 **qRT-PCR internal control primers.** Maize 18s ribosome RNA gene was used as the internal control to normalize the qRT-PCR results.
(XLSX)

Table S8 **miRNA targets qRT-PCR primers.** The first column is the miRNA id. The second column is the target gene id. The third column is the gene specific forward primer for the qRT-PCR. The fourth column is the gene specific reverse primer for the qRT-PCR. The fifth column is the designed product size (around 200 bp). The last column is the targets annotation.
(XLSX)

Table S9 **5'RLM-RACE validation of the miRNA targets nested PCR primers.** The first column is the miRNA id. The second column is the target gene id. The third column is the gene specific outer primer for the PCR. The fourth column is the gene specific inner primer for the PCR. The fifth column is the designed product size. The last column is the targets annotation. 5′RLM-RACE adapter is the adapter used for the 5′RACE analysis. 5′RACE outer primer is the universal outer primer for the PCR. 5′RACE inner primer is the universal inner primer for the PCR.
(XLSX)

Table S10 **Mapping of intragenic hypoxia responsive miRNAs with B73 maize genome.** Out of 64 differentially expressed miRNA genes representing 22 known mature miRNA signatures, 30 miRNA genes belonging to 13 miRNA families could be mapped to the transcription start site (TSS).
(XLSX)

Table S11 **Brief description of the list of transcription Factors from JASPAR Core Plantae whose binding sites were found on miRNA promoter region.** The first column is the TF id. The second column is the TF family. The third column is the TF class. The fourth column is the plant species for that TF annotated in JASPER. The fifth column is the reference.
(XLSX)

Table S12 **List of transcription factors that have been used for motifs predictions for probable TF-miRNA interactions for upstream analysis of miRNA-mediated gene regulatory sub-network.** The first column is the miRNA id. The second column is the TF that bind to the miRNA promoter. The third column is the TF family. The fourth column is the description of the TF family.
(XLSX)

Table S13 **List of miRNA targets that have been used for miRNA-TF interactions for downstream analysis of probable miRNA-mediated gene regulatory sub-network.** The first column is the miRNA id. The second column is the TF family of the miRNA targets. The third column is the description of the TF family.
(XLSX)
