# Supplementary figures and images for: Characterization of miRNAs in Response to Short-Term Waterlogging in Three Inbred Lines of Zea mays
Source: PLoS One. 2012 Jun 29;7(6):e39786. doi: 10.1371/journal.pone.0039786 (PMC3387268; doi:10.1371/journal.pone.0039786)

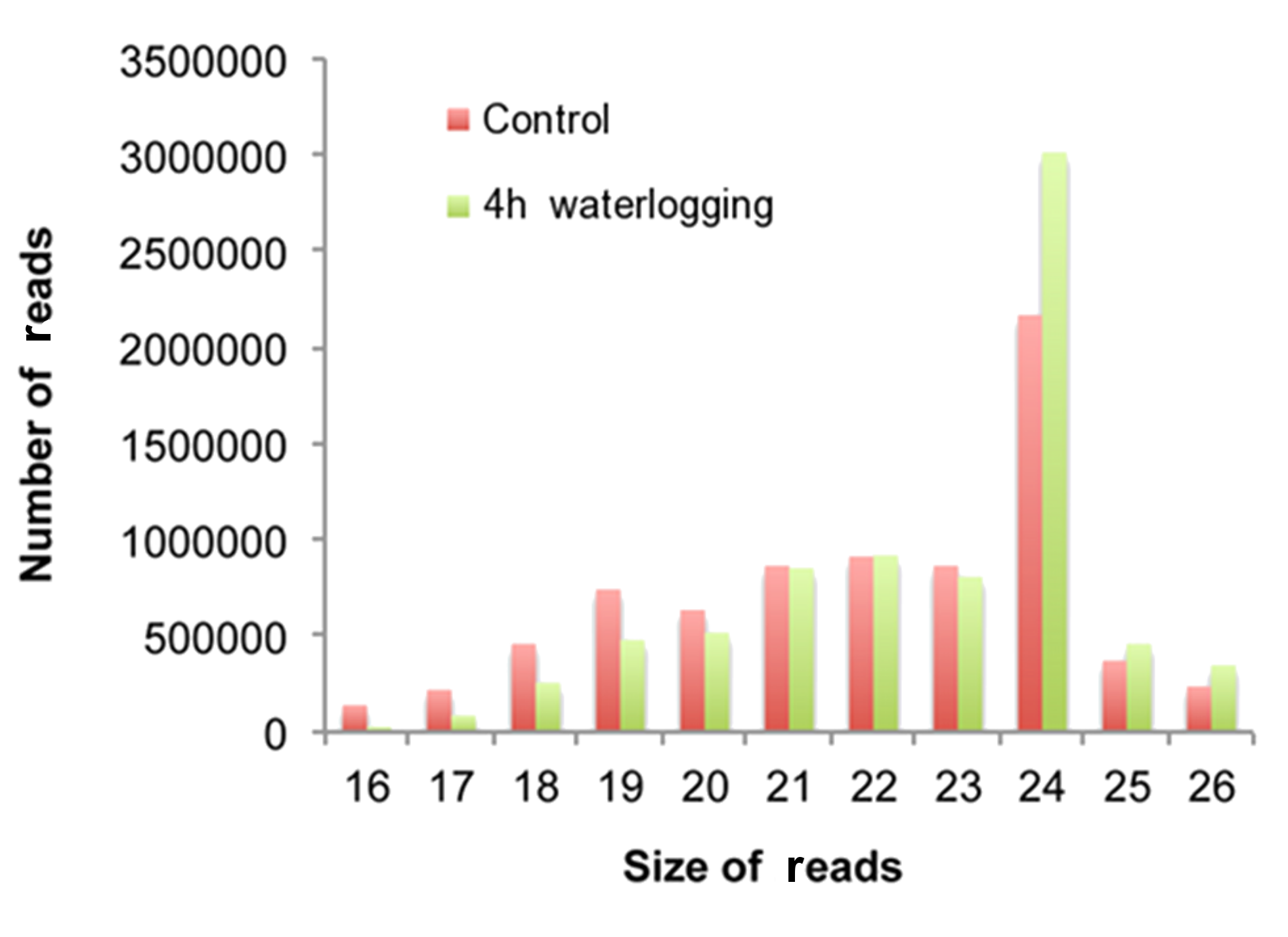

Supplement: Figure S1 — Size distribution of sequenced reads of control and 4 h waterlogging treated roots in Hz32. X-axis shows the size of the reads of the sequencing fragment. Y-axis shows the raw reads of each fragment. Red bar shows the results of control sample. Green bar shows the results of 4 h treatment sample. (TIF) [file pone.0039786.s001.tif]

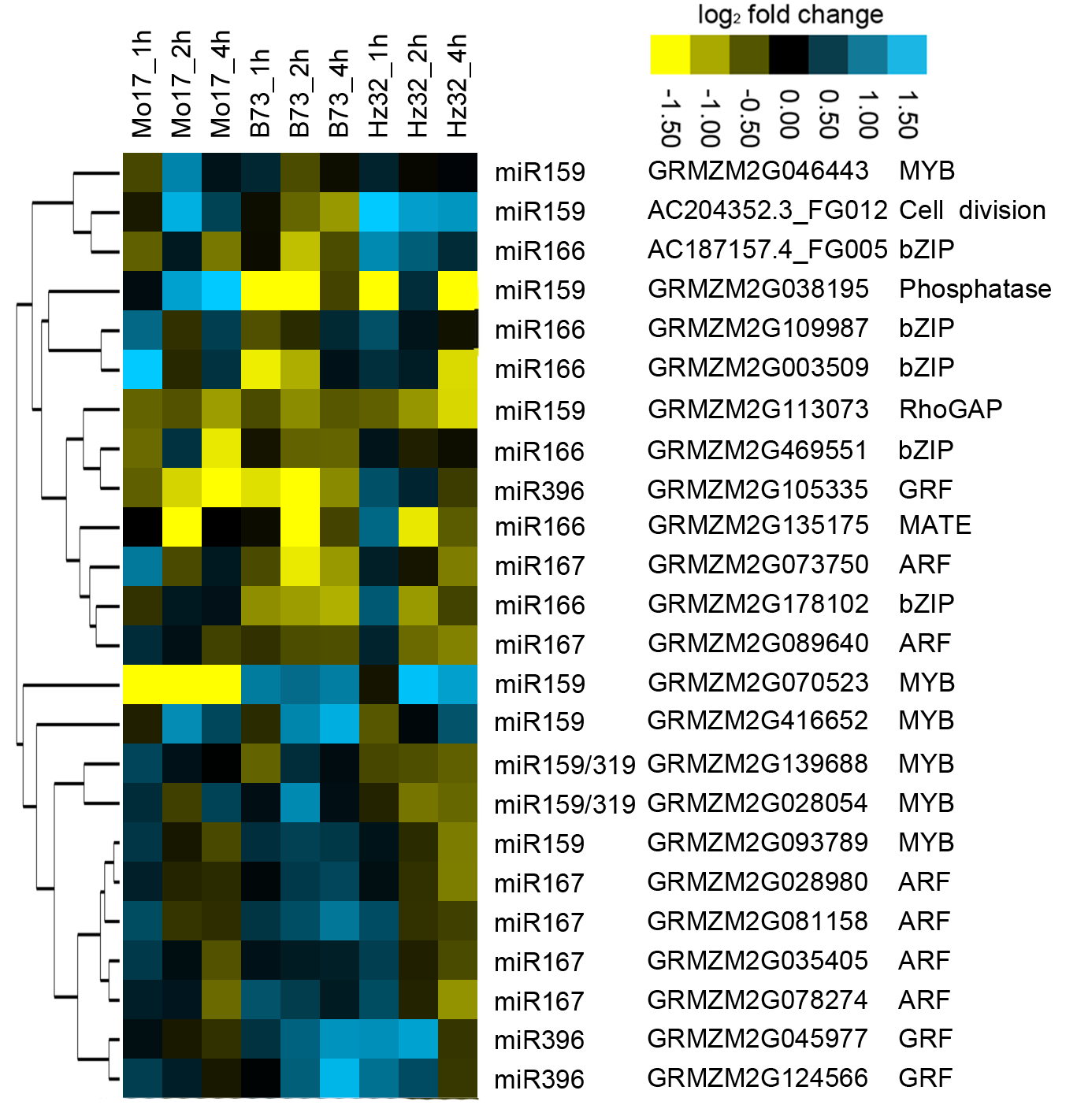

Supplement: Figure S2 — Cluster 1 miRNA targets expression profile. The cluster was done on the basis of log2 (expression level in treatment/expression level in control). Yellow color shows down-regulation. Blue color shows up-regulation. (TIF) [file pone.0039786.s002.tif]

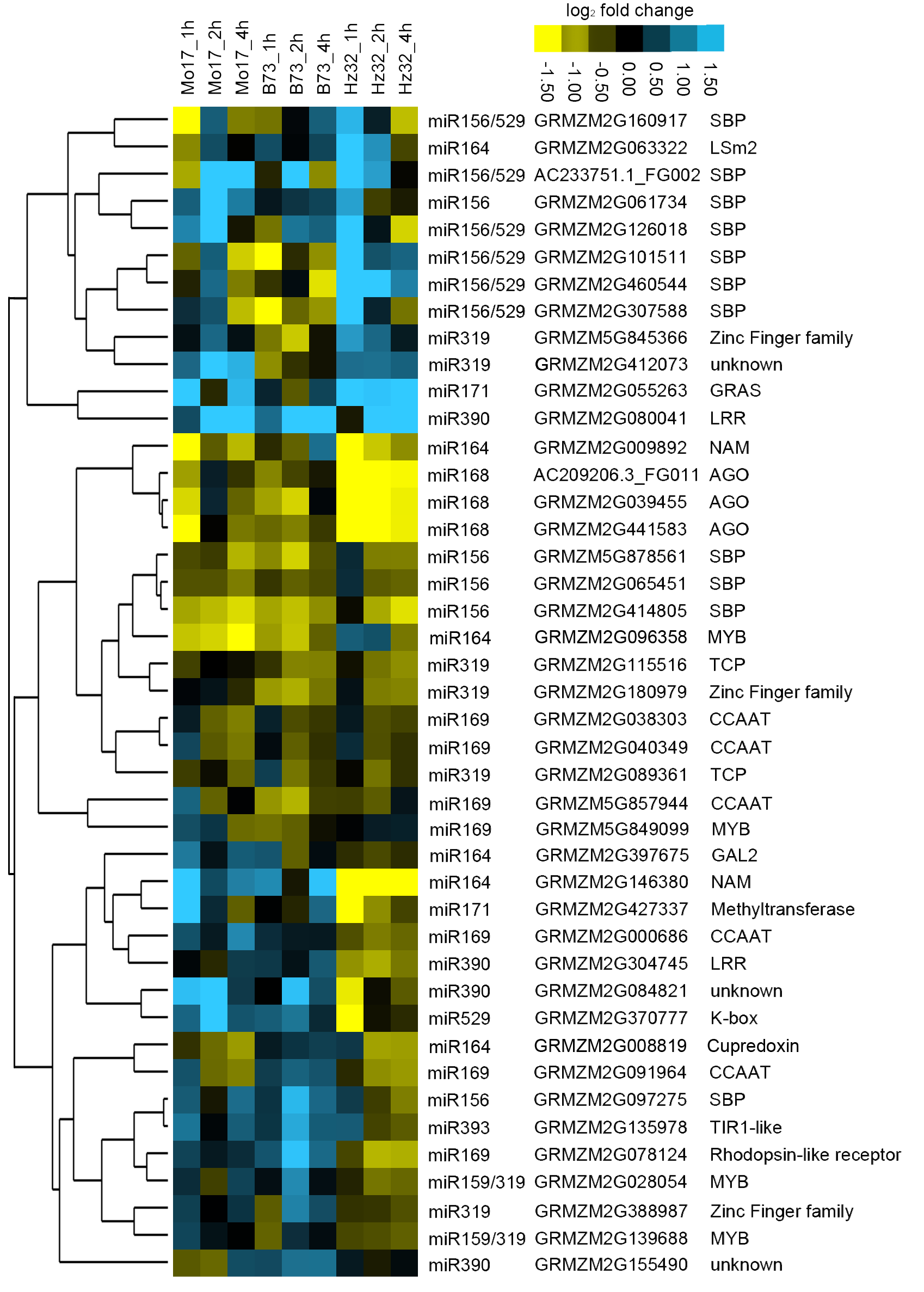

Supplement: Figure S3 — Cluster 2 miRNA targets expression profile. The cluster was done on the basis of log2 (expression level in treatment/expression level in control). Yellow color shows down-regulation. Blue color shows up-regulation. (TIF) [file pone.0039786.s003.tif]

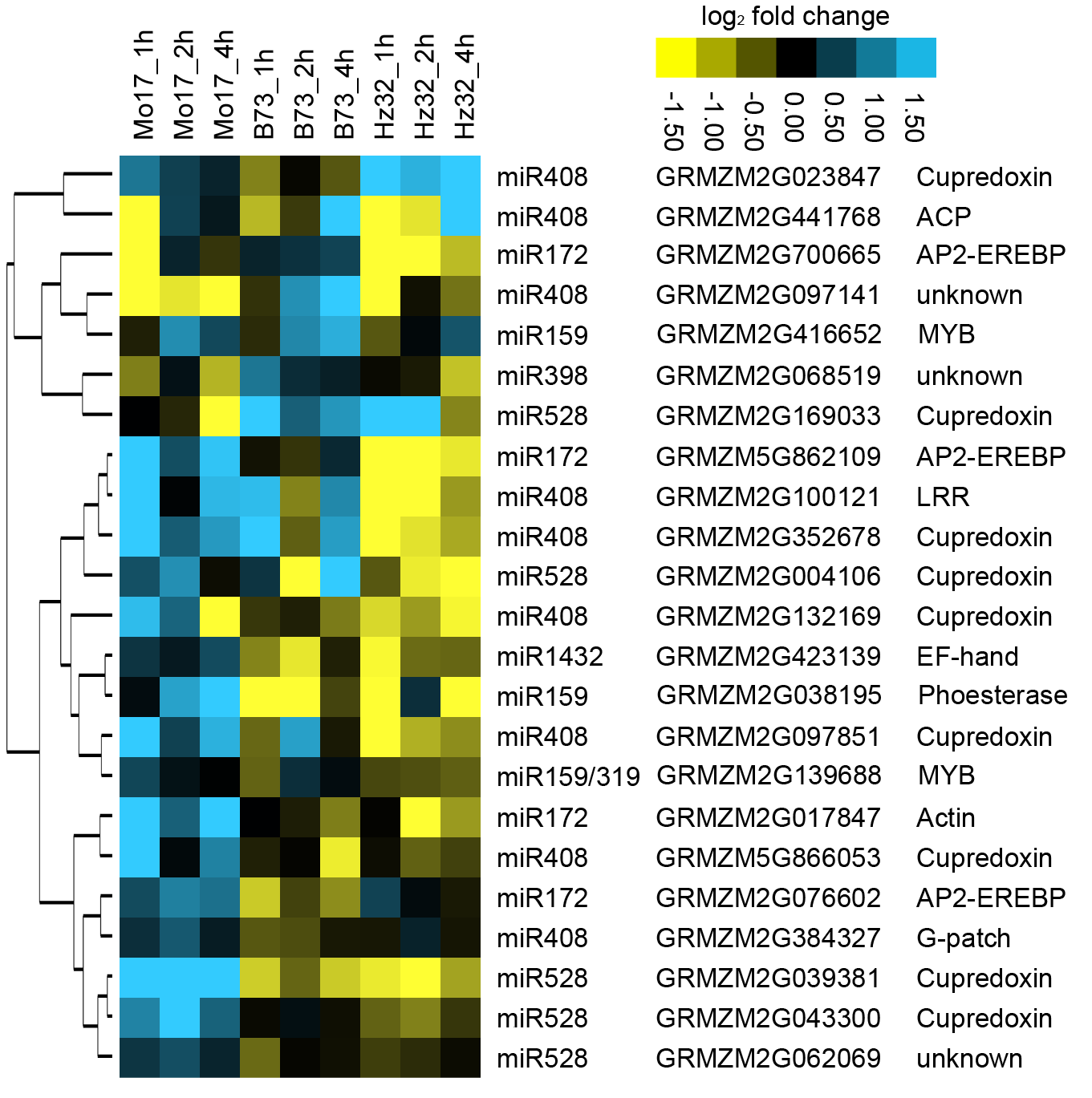

Supplement: Figure S4 — Cluster 3 miRNA targets expression profile. The cluster was done on the basis of log2 (expression level in treatment/expression level in control). Yellow color shows down-regulation. Blue color shows up-regulation. (TIF) [file pone.0039786.s004.tif]

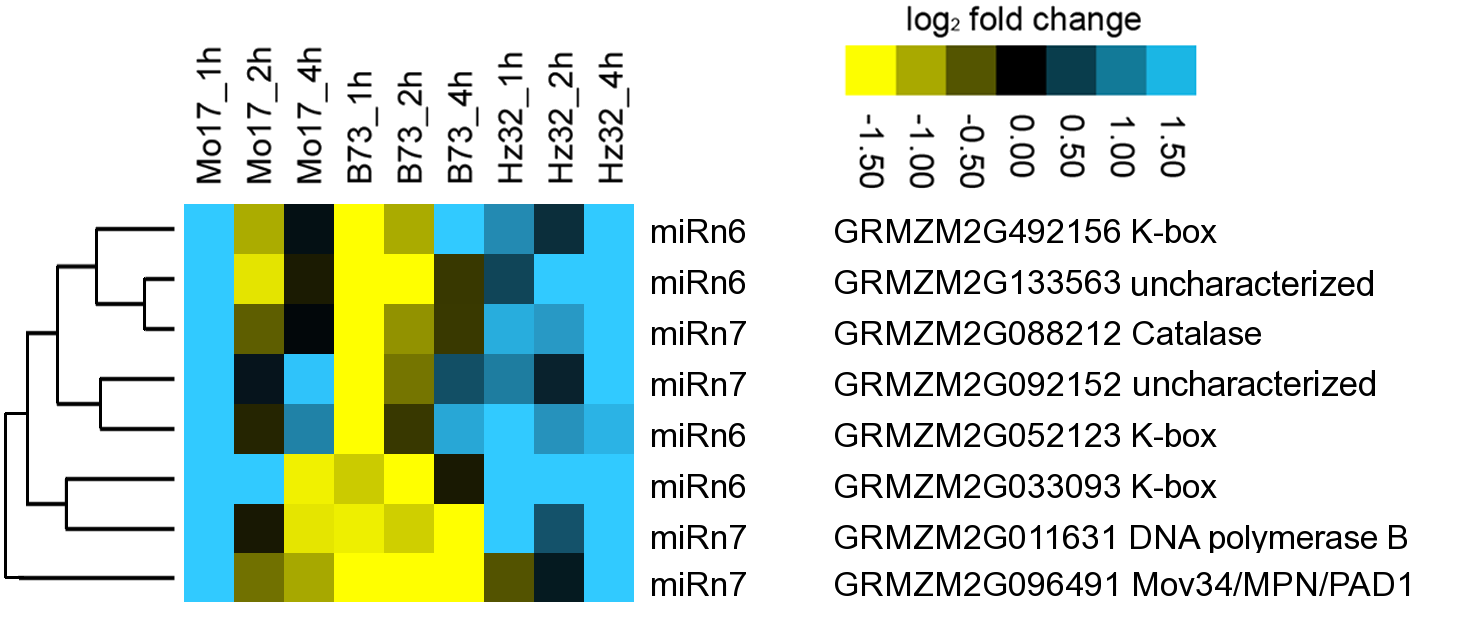

Supplement: Figure S5 — Cluster of novel miRNA targets expression profile. The cluster was done on the basis of log2 (expression level in treatment/expression level in control). Yellow color shows down-regulation. Blue color shows up-regulation. (TIF) [file pone.0039786.s005.tif]
